# Impact of universal access to hepatitis C therapy on HIV-infected patients: implementation of the Spanish national hepatitis C strategy

**DOI:** 10.1007/s10096-016-2822-6

**Published:** 2016-10-27

**Authors:** A. Rivero-Juarez, L. F. Lopez-Cortes, M. Castaño, D. Merino, M. Marquez, M. Mancebo, F. Cuenca-Lopez, P. Jimenez-Aguilar, I. Lopez-Montesinos, S. Lopez-Cardenas, A. Collado, M. A. Lopez-Ruz, M. Omar, F. Tellez, X. Perez-Stachowski, J. Hernandez-Quero, J. A. Girón-Gonzalez, E. Fernandez-Fuertes, A. Rivero, on behalf of the HERACLES cohort study team of the Grupo de Estudio de Hepatitis Virales (HEPAVIR) of the Sociedad Andaluza de Enfermedades Infecciosas (SAEI)

**Affiliations:** 1 Unidad de Enfermedades Infecciosas, Hospital Universitario Reina Sofía de Córdoba, Instituto Maimonides de Investigación Biomédica de Córdoba (IMIBIC), Universidad de Córdoba (UCO), Avenida Menéndez Pidal s/n, 14004 Córdoba, Spain; 2 Unidad Clínica de Enfermedades Infecciosas, Microbiología y Medicina Preventiva, Instituto de Biomedicina de Sevilla (IBiS), Hospital Universitario Virgen del Rocío/CSIC/Universidad de Sevilla, Seville, Spain; 3 Unidad Clínica de Enfermedades Infecciosas, Hospital Regional Universitario Carlos Haya, Málaga, Spain; 4 Unidad Clínica de Enfermedades Infecciosas, Complejo Hospitalario Universitario de Huelva, Huelva, Spain; 5 Unidad Clínica de Enfermedades Infecciosas y Microbiología, Hospital Regional Universitario Virgen de la Victoria, Málaga, Spain; 6 Unidad Clínica de Enfermedades Infecciosas y Microbiología, Instituto de Biomedicina de Sevilla (IBiS), Hospital Universitario de Valme, Seville, Spain; 7 Unidad Clínica de Enfermedades Infecciosas, Hospital Universitario Puerto Real, Cádiz, Spain; 8 Unidad Clínica de Enfermedades Infecciosas y Microbiología, Hospital Universitario Virgen Macarena, Seville, Spain; 9 Unidad Clínica de Enfermedades Infecciosas y Microbiología, Hospital de Jerez, Jerez, Spain; 10 Unidad Clínica de Enfermedades Infecciosas, Complejo Hospitalario Torrecárdenas, Almería, Spain; 11 Unidad Clínica de Enfermedades Infecciosas y Microbiología, Hospital Universitario Virgen de las Nieves, Granada, Spain; 12 Unidad Clínica de Enfermedades Infecciosas, Complejo Hospitalario de Jaén, Jaén, Spain; 13 Unidad Clínica de Enfermedades Infecciosas y Microbiología, Hospital La Línea, AGS Campo de Gibraltad, Cádiz, Spain; 14 Unidad de Medicina Interna, Hospital Costa del Sol, Marbella, Spain; 15 Unidad Clínica de Enfermedades Infecciosas, Hospital Universitario San Cecilio, Granada, Spain; 16 Unidad Clínica de Enfermedades Infecciosas y Microbiología, Hospital Puerta del Mar, Cádiz, Spain; 17 Unidad de Medicina Tropical, Hospital de Poniente, El Ejido, Spain

## Abstract

In April 2015, the Spanish National Health System (SNHS) developed a national strategic plan for the diagnosis, treatment, and management of hepatitis C virus (HCV). Our aim was to analyze the impact of this on human immunodeficiency virus (HIV)-infected patients included in the HERACLES cohort during the first 6 months of its implementation. The HERACLES cohort (NCT02511496) was set up in March 2015 to evaluate the status and follow-up of chronic HCV infection in patients co-infected with HIV in the south of Spain. In September 2015, the data were analyzed to identify clinical events (death, liver decompensation, and liver fibrosis progression) and rate of treatment implementation in this population. The study population comprised a total of 3474 HIV/HCV co-infected patients. The distribution according to liver fibrosis stage was: 1152 F0–F1 (33.2 %); 513 F2 (14.4 %); 641 F3 (18.2 %); 761 F4 (21.9 %); and 407 whose liver fibrosis was not measured (12.3 %). During follow-up, 248 patients progressed by at least one fibrosis stage [7.1 %; 95 % confidence interval (CI): 6.3–8 %]. Among cirrhotic patients, 52 (6.8 %; 95 % CI: 5.2–8.9 %) developed hepatic decompensation. In the overall population, 50 patients died (1.4 %; 95 % CI: 1.1–1.9 %). Eight hundred and nineteen patients (23.56 %) initiated interferon (IFN)-free treatment during follow-up, of which 47.8 % were cirrhotic. In our study, during 6 months of follow-up, 23.56 % of HIV/HCV co-infected patients included in our cohort received HCV treatment. However, we observed a high incidence of negative short-term outcomes in our population.

## Introduction

The high efficacy and safety of direct-acting antiviral drugs (DAAs) used for the treatment of hepatitis C virus (HCV) has meant a significant improvement in cure rates [defined as sustained virological response (SVR)] and, as a direct result, an improved prognosis for this infectious disease [1–3]. Nevertheless, incorporating DAAs into patient care requires implementing new and expensive resources, so limiting their application in countries like Spain, which have universal free healthcare systems. In this regard, in April 2015, the Spanish National Health System (SNHS) developed a strategic plan for diagnosing, treating, and managing HCV throughout Spain [4]. This plan was approved in April 2015 and extended across four strategic action lines, each with different objectives and key action points. These lines included: (i) quantifying the magnitude of this disease; (ii) defining the clinical criteria for implementing appropriate treatment; (iii) establishing coordination mechanisms to guarantee the proper implementation of the plan; and (iv) promoting knowledge about the prevention, diagnosis, and treatment of HCV by fomenting clinical and basic research [4]. To support these lines, key clinical criteria for starting HCV therapy were determined, as a result of which implementation of therapy was obligatory for the majority of patients (Table 1). The development and implementation of this plan, therefore, constitutes a major advance in the eradication of HCV in Spain.

**Table 1:** Criteria for treatment initiation and prioritization of the Spanish national strategic plan for the diagnosis, treatment, and management of HCV infection

Prioritization criteria for implementing HCV treatment
• All patients with liver fibrosis stages F4, F3, or F2
• Patients on the waiting list for liver transplant
• Patients with liver transplant, independent of stage of liver fibrosis
• Patients with failed treatment with Peg-IFN/RBV in combination with boceprevir or telaprevir
• Patient who are recipients of non-liver transplants, independent of stage of liver fibrosis
• Patients with extra-hepatic manifestations, independent of stage of liver fibrosis
• Patients with stage F0–F2 liver fibrosis, in the following situations:
• Patients at risk of transmission
• Women wishing to become pregnant

*HCV* hepatitis C virus; *F4* liver cirrhosis; *F3* liver fibrosis stage 3; *F2* liver fibrosis stage 2; *F1* liver fibrosis stage 1; *F0* absence of liver fibrosis; *Peg-IFN/RBV* pegylated interferon plus ribavirin

HCV patients co-infected with human immunodeficiency virus (HIV) form an especially sensitive population. The use of highly active antiretroviral therapy (HAART) led to liver disease, mainly HCV infection, being identified as the main cause of morbidity and mortality among HIV-infected patients [5]. This effect is a direct consequence of higher and faster liver fibrosis progression rates and lower sustained virological response (SVR) rates, even using interferon (IFN)-free treatments [6–8]. For these reasons, it is vitally important for this population of patients to be part of a national strategy.

There are very few reports showing the impact of a national program for treating chronic hepatitis C in HIV/HCV co-infected patients. Here, therefore, we analyzed the impact on HIV-infected patients included in the HERACLES cohort of the Spanish national strategic plan for diagnosing, treating and managing HCV infection during the first 6 months of its implementation.

## Methods

### Study design and population

The HERACLES cohort, “Status of Chronic Liver Disease in Hepatitis C Virus (HCV) Patients Coinfected With Human Immunodeficiency Virus (HIV) in Andalusia”, is a multicenter prospective observational cohort study of HIV-infected patients with active HCV co-infection (clinicaltrials.gov identification: NCT02511496). The cohort was set up in March 2015, with the main objective of evaluating the current status and follow-up of chronic HCV infection in patients co-infected with HIV in Andalusia (southern Spain).

This cohort included HIV-infected patients with chronic HCV infection in follow-up at 19 reference centers in Andalusia for the care of HIV-infected patients. These 19 centers look after a population of 15,556 HIV-infected patients, who represent 99.3 % of HIV-infected patients attending centers in the Andalusia healthcare system. These data were obtained after an epidemiological inquiry in which all HIV-infected patients attending the 21 reference centers of Andalusia (*n* = 15,663) were tested [9]. HIV-infected patients were included in the cohort if they matched the following inclusion criteria: (i) active chronic HCV infection (defined as detectable HCV RNA in serum) and (ii) not receiving treatment for HCV at inclusion.

### Variable collection and definition

At inclusion in the cohort (March–April 2015), the demographic, clinical, and virological characteristics of participants were collected and recorded. These were considered the baseline characteristics, and included: age, gender, risk for HCV infection, use of HAART, CD4+ cell count (cells/mL), plasma HIV viral load (copies/mL), acquired immune deficiency syndrome (AIDS)-defining criteria, HCV plasma viral load (IU/mL), HCV genotype/subtype, liver fibrosis stage, Child–Turcotte–Pugh (CTP) classification (A, B, or C), and history of HCV therapy.

Bi-annual follow-up of patients included in the cohort was planned. Variables collected and recorded at each follow-up visit included: liver fibrosis stage, use of DAA-based anti-HCV therapy during follow-up, achieving SVR, presence and type of liver decompensation, inclusion on the waiting list for transplant, liver transplant, death, and cause of death. Variables for this report were collected in August–September 2015 (6-month follow-up).

Liver fibrosis staging was performed by liver biopsy (following the METAVIR fibrosis score) and/or liver transient elastography (FibroScan; Echosens, Paris, France). Classification of liver fibrosis staging was by histological analysis or liver stiffness measurement (LSM) score as follows: (i) F0–F1 METAVIR fibrosis score or LSM <7.2 kPa; (ii) F2 METAVIR fibrosis score or LSM 7.2–8.9 kPa; (iii) F3 METAVIR fibrosis score or LSM 9–14.5; and (iv) F4 METAVIR fibrosis score or LSM ≥14.6 kPa. Liver fibrosis stage was measured at baseline and at each subsequent visit in order to determine the liver fibrosis stage at each visit and, hence, the degree of liver fibrosis progression. A liver fibrosis stage of at least one stage higher with respect to the baseline was considered liver fibrosis progression. Liver stiffness measurement was planned for each study visit.

In cirrhotic patients, liver decompensation was considered as the following hepatic events: portal hypertensive gastrointestinal bleeding (PHGB), ascites, hepatorenal syndrome (HRS), spontaneous bacterial peritonitis (SBP), hepatic encephalopathy (HE), and hepatocarcinoma (HCC).

Health status or cause of death was collected at each visit. Cause of death was classified as AIDS-related, liver-related, or other cause. Exitus due to other causes was specified in the data collection.

HIV and HCV viral loads were obtained using reverse transcription polymerase chain reaction (RT-PCR) (Cobas TaqMan; Roche Diagnostic Systems Inc., Pleasanton, CA, USA). Undetectable HIV viral load was defined as an HIV RNA viral load of less than 50 copies per mL.

### Statistical analysis

Analysis of the population included in the study was descriptive. Characteristics of patients (qualitative variables) were reported as the number of cases (percentages). Numerical variables were reported as medians (interquartile range). Otherwise, the population description was divided into patients who were receiving HCV treatment during follow-up and those who were not. For patients receiving treatment during follow-up, a comparative analysis according to HCV genotype, liver fibrosis stage, and previous experience of HCV therapy was performed in order to identify variables with an impact on the clinical decision to initiate therapy. Those variables were compared using the Chi-square test.

An analysis evaluating the rate of liver fibrosis progression was performed. Liver fibrosis progression was considered as an increase of at least one liver fibrosis stage with respect to the baseline. Patients with liver fibrosis stage F4 at baseline were censored from analysis. The mortality rate was calculated as the overall number of deaths in the total population. Otherwise, mortality rates were determined for patients with cirrhosis (F4) and F0–F3 patients, specifying cause of death.

### Ethics

This study was conducted according to the principles of Good Clinical Practice (Ministry of Health, Royal Decree 223/2004 of 6 February) and the Declaration of Helsinki. The study did not require informed consent because the patients were not directly interviewed and completely anonymous information was collected from existing records, ensuring the protection of personal data in accordance with Law 15/1999 of 13 December on Personal Data Protection. The study coordinator presented the study protocol (protocol code: FIBICO-0015/01017-2015) to the Coordinating Ethics Committee for Biomedical Research in Andalusia for evaluation and obtained approval (240-2819-05/15). Those responsible for the provision of health services where the study occurred were given a copy of the protocol and documents evidencing approval by the Ethics Committee in accordance with the procedures and legal requirements.

## Results

### Baseline population and characteristics

A total of 3474 HIV/HCV co-infected patients were included in the cohort and formed the study population. This implies a prevalence of active HCV infection in the south of Spain of 22.4 %. The distribution of patients according to HCV genotype was: 1944 HCV genotype 1 (56.07 %), 38 HCV genotype 2 (1.01 %), 559 HCV genotype 3 (16.08 %), 736 HCV genotype 4 (21.2 %), three HCV genotype 6 (0.08 %), and 194 whose HCV genotype was not available (5.56 %). Among patients infected by HCV genotype 1, 862 were genotype 1a (44.2 %), 461 genotype 1b (23.6 %), 49 HCV genotype 1ab (2.6 %), and in 572 patients, the HCV subtype was not determined (29.6 %). When patients were sorted according to liver fibrosis stage, 1152 patients were F0–F1 (33.2 %), 513 were F2 (14.4 %), 641 were F3 (18.2 %), 761 were F4 (21.9 %), and in 407 patients, liver fibrosis was not staged (12.3 %). When patients were grouped according to previous experience of HCV therapy, 2453 were naïve (70.6 %), 898 did not respond to pegylated interferon plus ribavirin (Peg-IFN/RBV) (25.8 %), and 123 did not respond to Peg-IFN/RBV in combination with boceprevir or telaprevir (3.6 %).

### Follow-up: clinical events

After 6 months of follow-up, liver fibrosis stage had progressed by at least one stage in 248 patients [7.1 %; 95 % confidence interval (CI): 6.3–8 %]. Of these, 108 F0–F1 patients progressed to F2–F4 (9.3 %; 95 % CI: 7.8–11.2 %), 80 F2 patients progressed to F3–F4 (15.6 %; 95 % CI: 12.7–19 %), and 59 F3 patients progressed to F4 (9.2 %; 95 % CI: 7.2–11.7 %).

Among 761 patients with cirrhosis, 52 (6.8 %; 95 % CI: 5.2–8.9 %) developed hepatic decompensation during the 6 months of follow-up, which included ascites in 28 (53.8 %) patients, HCC in 14 (26.9 %), PHGB in 6 (11.5 %), HE in 3 (5.7 %), and SBP in 1 (1.9 %). The cumulative incidence of HCC in cirrhotic patients during the 6 months of follow-up was 1.8 % (95 % CI: 1.1–3.1 %).

Fifty patients died during follow-up. The mortality rate was 1.4 % (95 % CI: 1.1–1.9 %). Causes of death according to liver fibrosis stage are summarized in Table 2.

**Table 2:** Causes of death during follow-up, according to liver fibrosis stage

	Overall (*n* = 3474)	F0–F3 (*n* = 2713)	F4 (*n* = 761)	*p*-Value
Death (*n* = 50)	50 (1.4 %)	34 (1.2 %)	16 (2.1 %)	0.082
Causes	*n* =50	*n* = 34	*n* = 16	
Liver-related	15 (30)	4 (11.7 %)	11 (68.7)	<0.001
Non-AIDS malignancies	10 (19.2)	6 (17.6 %)	4 (25)	0.588
Systemic bacterial infection	7 (13.4)	7 (20.6 %)	0	0.054
Cardiovascular event	6 (9.6)	6 (17.6 %)	0	0.116
Abuse of toxic substances	4 (7.6)	3 (8.8 %)	1 (6.3)	0.792
AIDS-related	3 (5.7)	3 (8.8 %)	0	0.389
Gastrointestinal bleeding*	2 (3.8)	2 (5.9 %)	0	0.771
End-stage renal disease	2 (3.8)	2 (5.9 %)	0	0.771
Suicide	2 (3.8)	1 (3.1 %)	0	0.66

*Gastrointestinal bleeding does not include bleeding from esophageal varices

### Follow-up: treatment implementation

Eight hundred and nineteen patients (23.56 %) initiated treatment for HCV infection in the first 6 months of the implementation of a national strategy for the diagnosis, treatment, and management of HCV in Spain, or a rate of 4.55 treatment implementations per day. 2654 patients, therefore, were not treated. The main baseline characteristics of treated and non-treated patients are shown in Table 3. Of the 52 patients in the decompensated liver stage of F4, 50 initiated therapy against HCV infection. The two patients who did not initiate therapy died before starting treatment due to complications of liver decompensation.

**Table 3:** Characteristics of treated and non-treated patients

Characteristics	Treated (*n* = 819)	Non-treated (*N* = 2654)	*p*-Value
Age (years), median (IQR)	50 (47–53)	48 (45–52)	0.001
Gender, no. (%)			
Male	683 (83.3)	2437 (91.7)	0.256
Female	136 (16.7)	217 (8.3)	
Risk group for HCV infection, no. (%)			
IDU	710 (86.7)	2318 (87.3)	0.876
Sexual	97 (11.8)	321 (12.1)	
Blood-derived	12 (1.5)	15 (0.6)	
HAART, no. (%)			
Receiving	810 (98.9)	2550 (96)	0.214
Non-receiving	9 (1.1)	104 (4)	
CD4+ total count (cells/mL), median (IQR)	527 (344–760)	487 (305–702)	0.001
HCV genotype, no. (%)			
1	508 (62.02)	1436 (54.2)	0.72
1a	253 (49.8)	609 (42.2)	
1b	116 (22.8)	345 (23.9)	
1a/b	16 (3.2)	33 (2.3)	
NG	123 (24.2)	449 (31.9)	
2	10 (1.2)	28 (0.9)	
3	131 (15.9)	428 (16.1)	
4	169 (20.6)	567 (21.3)	
6	1 (0.28)	2 (0.2)	
NG	0	194 (7.3)	
Liver fibrosis stage, no. (%)			
F0–F1	90 (10.9)	1062 (40)	<0.001
F2	79 (9.6)	434 (16.3)	
F3	222 (27.1)	419 (15.7)	
F4	392 (47.8)	369 (13.9)	
Not staged	36 (4.6)	371 (14.1)	
Previous experience of HCV therapy, no. (%)			
Naïve	366 (44.6)	2087 (78.6)	0.014
Failure of Peg-IFN/RBV	373 (45.5)	525 (19.7)	
Failure of BOC- or TPV-based regimen	80 (9.9)	43 (1.7)	

*IQR* interquartile range; *IDU* injecting drug user; *HAART* highly active antiretroviral therapy; *NG* non-genotyped; *BOC* boceprevir; *TPV* telaprevir

**Fig. 1:**
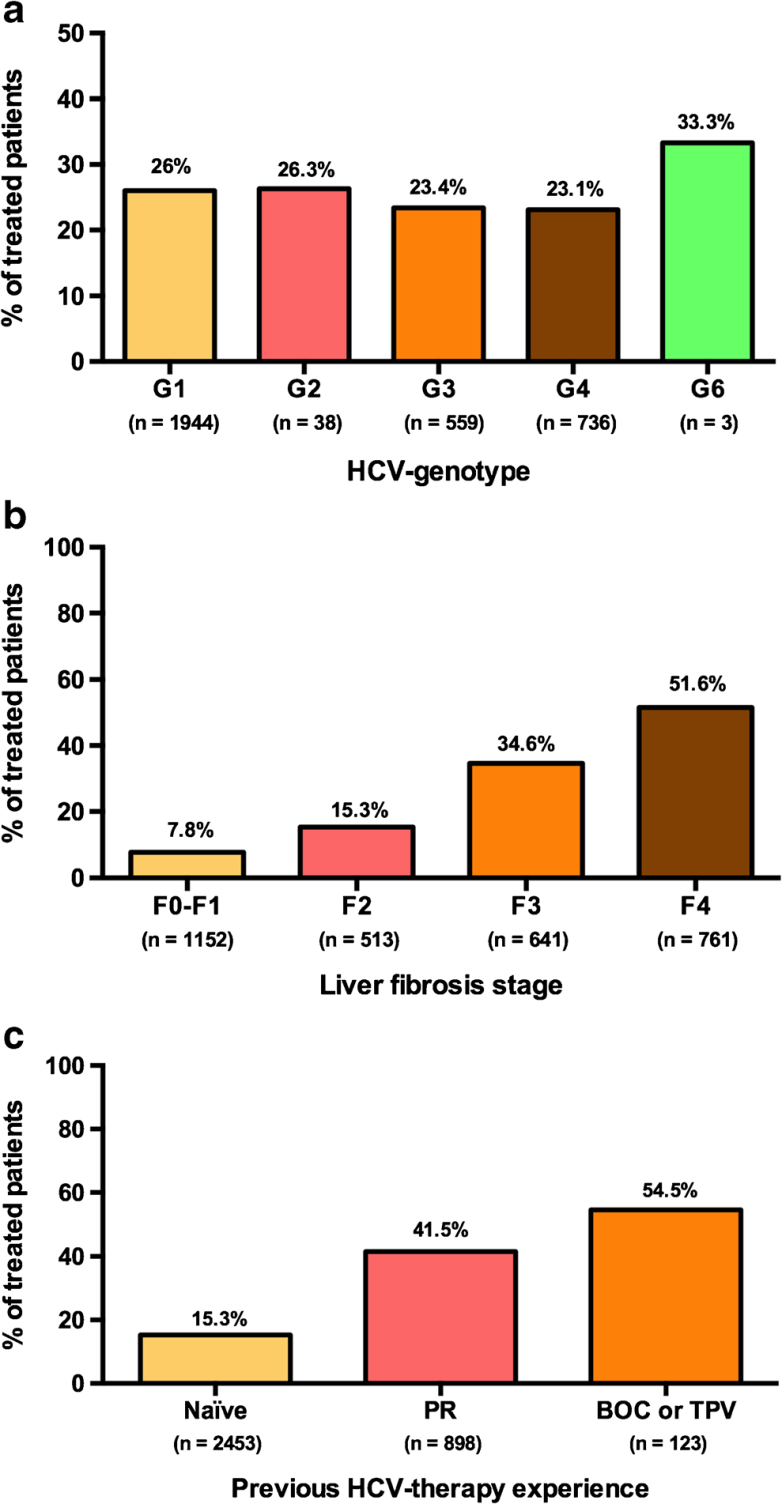
Distribution of patients receiving hepatitis C virus (HCV) therapy, according to HCV genotype (**a**), liver fibrosis stage (**b**), and previous experience of HCV therapy (**c**), with respect to the global population

The proportion of treated patients by liver fibrosis stage, HCV genotype, and previous experience of HCV therapy are shown in Fig. 1. A total of 90 F0–F1 patients initiated treatment during the follow-up. Of these, 49 (54.4 %) experienced treatment failure with a DAA + Peg-IFN/RBV regimen, 27 (28.8 %) showed extra-hepatic manifestations or another comorbidity, 13 (14.4 %) were treated for epidemiological reasons, and 1 (2.4 %) was a renal transplant patient.

## Discussion

We report the first results of the impact of the Spanish national strategic plan for the diagnosis, treatment, and management of HCV on HIV/HCV co-infected patients in the HERACLES cohort. During the first 6 months of follow-up, a high proportion (819, 23.56 %) of HIV/HCV co-infected patients included in the cohort initiated DAA-based anti-HCV therapy. However, despite this, there was a high incidence of negative short-term outcomes among the HIV/HCV patients in our cohort; in this regard, 248 (7.1 %; 95 % CI: 6.3–8 %) patients progressed by at least one stage of liver fibrosis, 52 cirrhotic patients (6.8 %; 95 % CI: 5.2–8.9 %) developed hepatic decompensation, and 50 patients (1.4 %; 95 % CI: 1.1–1.9 %) died.

Liver fibrosis stage was the main factor involved in the decision to initiate anti-HCV therapy. Cirrhotic patients constitute the principal sensitive HCV population group due to the short-term risk of liver decompensation and liver-related death [10, 11]. The benefit of successful anti-HCV treatment in this subset of patients, in the sense that it significantly reduces both morbidity and mortality, is generally recognized [3, 12]. The general use of direct-acting antiviral agents is, therefore, a priority for these patients because this is the target population that derives the most benefit in the short term [13]. In our cohort, 761 patients (24.8 %) showed liver cirrhosis and 392 (51.5 %) of these received anti-HCV therapy during the 6 months of follow-up. By contrast, 52 (6.8 %) cirrhotic patients suffered liver decompensation during follow-up and 15 (1.9 %) died. On the other hand, the number of active HCV infection patients in our cohort with no or mild liver fibrosis (F0–F2) starting treatment for hepatitis C was low. Stage F0–F1 fibrosis was detected in 1152 (33.1 %) patients, and 90 (7.8 %) of these received anti-HCV therapy. This proportion was significantly lower than for stage F2 (15.3 %), stage F3 (34.6 %), and cirrhotic patients (51.5 %), most likely due to prioritization policies in the prescription of IFN-free, direct-acting antiviral agent-based therapies for HCV in the Spanish national plan. In addition, HIV/HCV co-infected patients represent a highly sensitive population due to the high rate of liver fibrosis progression compared to HCV monoinfected patients [14, 15]. Indeed, in our cohort, 8 % of F0–F2 patients progressed to the F3–F4 stage of liver fibrosis during just 6 months of follow-up. This observation may be added to the list of reasons in favor of prioritizing anti-HCV treatment, regardless of fibrosis stage, in patients with HIV/HCV co-infection.

Chronic HCV infection increases the mortality rate for hepatic and extra-hepatic diseases [16]. In our cohort, 52 (1.5 %) patients died during the 6 months of follow-up. Liver-related deaths accounted for 30 % of all causes of death in our study. The course of chronic HCV is accelerated in HIV/HCV-co-infected patients, with more rapid liver fibrosis progression and more frequent hepatic decompensation events compared to HCV monoinfected patients [7, 8, 17]. Consequently, HCV-related liver complications have emerged as a significant cause of mortality among HIV/HCV co-infected patients. HAART is an important factor in slowing liver fibrosis progression, although its use is not sufficient to reduce the rates of end-stage liver disease to those of HCV monoinfected patients [7]. For this reason, HIV co-infected patients are considered a priority for receiving anti-HCV therapy regardless of liver fibrosis stage, so as to reduce the rate of hepatic decompensation. As expected in a cohort of HIV-infected patients containing a high proportion of subjects with suppressed HIV viral loads due to HAART, mortality due to AIDS-related events was low, accounting for 5.7 % of causes of death. In contrast, non-AIDS/non-liver-related conditions constituted the main cause of death in our cohort. Cardiovascular diseases and non-AIDS/non-liver malignancies have been related to both HCV and HIV infection. In our cohort, non-AIDS/non-liver malignancies comprised 64.3 % of all causes of death. The incidence of non-AIDS-defining malignancies is elevated in chronic HIV infection [18, 19]. In addition, some evidence has suggested that HCV may be associated with the development of malignancies such as B-cell non-Hodgkin lymphoma, intrahepatic cholangiocarcinoma, and the incidence of pancreatic cancer [20]. Genomic and/or HCV replicative sequences have been detected in extra-hepatic localizations and it has been speculated that HCV may promote persistent inflammation in extra-hepatic organs and induce cancerous transformations [20]. Finally, in our cohort, five patients died due to cardiovascular disease; in other words, 9.6 % of all deaths. This is not striking, because cardiovascular disease is a major cause of morbidity and mortality in HIV-infected patients, accounting for at least 10 % of deaths [21, 22], and the evidence in favor of HCV infection promoting major risk factors for cardiovascular disease is compelling [23].

The prevalence of HCV active infection among HIV-infected patients has decreased in recent years. The prevalence of chronic HCV infection in Spain in 2002 was 54 % [24], decreasing to 34 % in 2009 [25], then to 22.1 % (402 out of 1882) in 2015 [26]. The baseline prevalence (March 2015) of chronic HCV infection in our cohort was similar, at 22.4 %. The progressive decrease of active HCV infection in our setting is due to at least three factors. Firstly, to changes in the route of HIV transmission. The decline of injecting drug users (IDUs) as the predominant route for transmission of HIV infection is probably the main factor governing the steady decrease in active HCV infection in Spain [27]. The prevalence of IDUs among Spanish patients newly infected with HIV decreased from 67.3 % in 1997 to 14.5 % in 2006, with a parallel reduction in the rate of HCV co-infection (from 73.8 % in 1997 to 19.8 % in 2006) in these patients [27]. Secondly, the reduction in active HCV infection is due to the high mortality among HIV/HCV co-infected patients. It is well established that individuals with HIV/HCV co-infection have significantly higher morbidity and mortality rates compared to individuals with HIV monoinfection [5, 28, 29]. As a result, liver-related complications currently represent one of the leading causes of death in the HIV-infected population. This particular aspect may have contributed to the progressive decrease in active HCV infection among HIV-infected patients in our area. Finally, the third reason is the use of highly effective anti-HCV therapy. The great majority of clinical trials evaluating the efficacy of DAAs have demonstrated high SVR rates in both HIV/HCV co-infected and HCV-infected patients [1, 2]. In our cohort, during the first 6 months of implementation of this national strategic plan for HCV, 819 (23.5 %) of the HIV/active HCV-infected patients received DAA-based anti-HCV therapy. It is assumed that, in the coming months, the widespread use of anti-HCV therapy will increase HCV cure rates and, hence, reduce active HCV infection rates among HIV/HCV co-infected individuals.

Our study presents several limitations that should be noted. Firstly, in the initiation phase of the study, not all DAA regimens had been approved in Spain and, so, the impact of this national strategic plan for the diagnosis, treatment, and management of HCV may be limited in this respect. Secondly, due to the fact that only HIV/HCV co-infected patients were included in our cohort, it was not possible to compare the impact of implementation on HCV monoinfected patients. Thirdly, liver fibrosis stage could not be established in 12.3 % of patients included in the study. These patients represent a special population for whom the national strategic plan cannot be implemented because of the absence of priority markers. Finally, although we mentioned that the majority (99.3 %) of HIV/HCV co-infected patients being followed in our area were included in our cohort, we do not know what proportion of patients have no access to clinical care.

In conclusion, during the first 6 months, 1 in 4 HIV/HCV co-infected patients, mainly with advanced liver fibrosis, were treated in the HERACLES cohort as a result of the Spanish national strategic plan for the diagnosis, treatment, and management of HCV. However, despite this apparent good news, the high incidence of negative short-term outcomes observed in our cohort makes it indispensable to continue providing extra resources to increase the rate of HIV/HCV co-infected patients receiving anti-HCV therapy, in order to improve the prognosis for this highly sensitive population.
